# An Integrated Mutually Oriented “Chemical Profiling–Pharmaceutical Effect” Strategy for Screening Discriminating Markers of Underlying Hepatoprotective Effects to Distinguish Garden-Cultivated from Mountain-Cultivated *Ginseng*

**DOI:** 10.3390/molecules26185456

**Published:** 2021-09-08

**Authors:** Saiyu Li, Yiwen Zhang, Panpan Yang, Minghui Tong, Luwen Xing, Qian Zhang, Kaishun Bi, Qing Li

**Affiliations:** School of Pharmacy, Shenyang Pharmaceutical University, Shenyang 110016, China; Saiyu_Li@outlook.com (S.L.); zzzyyywen@outlook.com (Y.Z.); Yang_panpan0112@163.com (P.Y.); tongminghui0930@163.com (M.T.); xlw_aikoaikko@163.com (L.X.); zhangqian@syphu.edu.cn (Q.Z.); kaishunbi.syphu@gmail.com (K.B.)

**Keywords:** garden-cultivated *Ginseng*, mountain-cultivated *Ginseng*, chemometric analysis, chemical profiling, hepatoprotective activity, discriminating markers

## Abstract

Garden-cultivated *Ginseng* (GG) and mountain-cultivated *Ginseng* (MG) both belong to *Panax Ginseng* C. A. Meyer. However, the effective substances which can be used to distinguish GG from MG remain obscure. Therefore, the purpose of this study was to screen for discriminating markers that can assist in the correct identification of GG and MG. HPLC Q-TOF/MS and various chemometrics methods were used to analyze the chemical profiles of 13 batches of *Ginseng* and to explore the characteristic constituents of both GG and MG. The hepatocyte-protecting effects of GG and MG were investigated through a paclitaxel-induced liver injury model. Through a combination of correlation analysis and bioinformatic techniques, markers for differentiation between GG and MG were ascertained. A total of 40 and 41 compounds were identified in GG and MG, respectively, and 15 characteristic ingredients contributed significantly to the discrimination of GG from MG. Correlation analysis and network pharmacology were applied and ginsenosides Rg1, Re, Rb1, Rc, Rb2, and Rg3 were found to be discriminating markers of GG and MG. Six markers for the identification of GG and MG were screened out by a step-wise mutually oriented “chemical profiling–pharmaceutical effect” correlation strategy, which is of great significance for future quality assessment of *Ginseng* products.

## 1. Introduction

*Panax Ginseng* C. A. Meyer (*Ginseng*), of genus Panax and family Araliaceae, has been used in traditional Chinese medicines (TCMs) for thousands of years throughout Asia to reinforce vital energy and tonify the spleen [1,2]. Based on extensive studies, *Ginseng* is rich in polysaccharides, ginsenosides, and volatile oils. More recently, numerous pharmacological studies have demonstrated that *Ginseng* possesses various biological activities, including antitumor, anti-inflammation, antioxidation, and immunoregulatory properties, among others [3,4,5]. Due to its diverse pharmacodynamics, *Ginseng* has been utilized in varied health products and dietary nutrients to relieve fatigue, deodorize, and ameliorate the toxic side effects of chemotherapy [6,7]. Recently, *Ginseng* has been clinically recognized as a hepatic protectant for its significant hepatoprotective effects and ability to improve liver function, as revealed by multitudinous studies [8,9,10,11].

Over recent years, inconsistency in the quality and therapeutic efficacy of available *Ginseng* products has become an intricate issue due to disparate growth environments and cultivation conditions. In the Chinese Pharmacopoeia (2020 edition), *Ginseng* was classified as either garden-cultivated *Ginseng* (GG, artificially cultivated) or mountain-cultivated *Ginseng* (MG, grown naturally under mountainous forest). In general, GG can be harvested after four to six years, while MG requires 10–20 years or longer. Both modern pharmacological research and traditional clinical experience indicate that the medicinal value and health function of MG is significantly better than that of GG [12,13]; thus, MG is consistently more expensive than GG. On account of the obvious differences in time investment and economic benefit, substitution of MG with GG has been increasingly rampant in the market, which is a primary cause of the current difficulty in maintaining the quality and effectiveness of *Ginseng* products. Thus, recent studies concerning the discrimination of GG from MG have attracted increasing attention. Previous research mainly focused on the contrastive qualitative-quantitative analysis of their chemical components in vitro [14,15,16]; however, it has been recognized that pharmaceutical efficacy is essentially the nucleus of TCM [17,18,19,20]. Unfortunately, few studies have sought to determine differentiating markers of GG and MG on the basis of their biological activity. Therefore, it is essential to integrate multiple analytical, pharmacological, and statistical approaches to establish a systematic strategy to explore the distinctive bioactive markers of GG and MG for their identification and differentiation.

In the present study, pharmacology-based markers for distinguishing GG from MG were first suggested by analyzing the chemical components of GG and MG using efficiency indexes. In brief, the ingredient database of *Ginseng* was obtained from seven batches of GG and six batches of MG analyzed in Liaoning by an established HPLC Q-TOF/MS method. Then, a variety of chemometric methods including hierarchical cluster analysis (HCA), principal component analysis (PCA), and orthogonal partial least squares discriminant analysis (OPLS-DA) were applied to explore the characteristic components of GG and MG. A contrastive study on the hepatoprotective function of GG and MG utilized a liver injury model induced by paclitaxel [21], which indicated that the liver-protective efficacy of MG was superior to that of GG. In order to further investigate the ingredients contributing to the different pharmacodynamics of GG and MG, we carried out a correlation analysis between the characteristic constituents and the efficiency indexes. Furthermore, in consideration of the “multi components-multi targets” characteristic of TCMs, network pharmacology was adopted to comprehensively screen for the effective discriminating markers of *Ginseng*. Above all, a step-wise mutually oriented “chemical profiling–pharmaceutical effect” strategy was established, and six chemical components—including ginsenosides Rg1, Re, Rb1, Rc, Rb2, and Rg3—were ascertained to be key markers, underlying the difference in hepatoprotective efficacy, for differentiating GG from MG. This study is of great significance in setting a more scientific benchmark for the analysis of *Ginseng,* and offers an alternative quality assessment system for TCMs.

## 2. Results and Discussion

### 2.1. Qualitative and Semi-Quantitative Analysis of Ingredients in Ginseng with HPLC Q-TOF/MS

**Figure 1:**
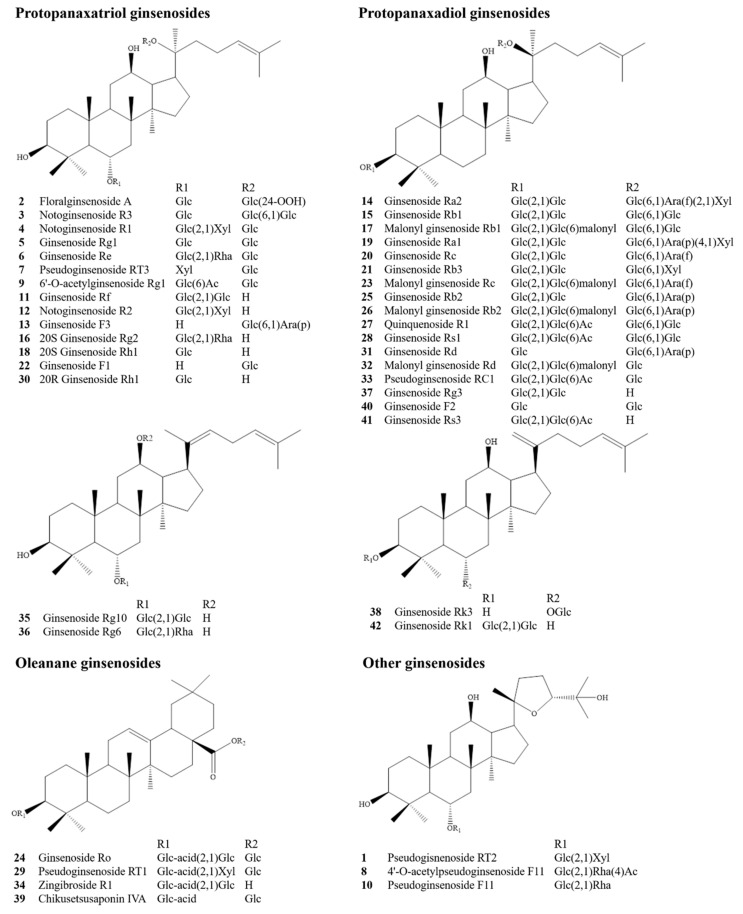
Chemical structures of the 42 components.

An established HPLC Q-TOF/MS method using digoxin as an internal standard (IS) was adopted to conduct the qualitative and semiquantitative analysis of the ingredients in *Ginseng* for seven batches of GG and six batches of MG, whose detailed information is presented in Table 1. The chemical components were identified by calculating possible chemical formulas based on comparisons between the determined molecular weight and chromatographic retention behavior and reference data [22,23,24]. As a result, a total of 42 chemical components were identified, including 19 protopanaxadiol ginsenosides (PPD-type), 16 protopanaxatriol ginsenosides (PPT-type), 4 oleanane ginsenosides, and 3 other ginsenosides; their structures are presented in Figure 1. A total of 40 and 41 compounds were identified in GG and MG, respectively, 40 of which were common to both, as shown in Table 2. Moreover, among them, 12 chemical ingredients were accurately identified by comparison with the reference substances (extraction ion chromatograms of reference substances and *Ginseng* sample solution are presented in Figure S1A–C).

### 2.2. Exploration of Characteristic Components for Differentiating GG from MG by Chemometric Analysis

Characteristic components for the identification of GG and MG were explored using multifarious chemometric analysis methods. Internal standard normalization (via digoxin) was adopted in this study. The peak areas of all 42 compounds were calibrated to the IS and the ratio of the peak areas were used for chemometric analysis.

**Figure 2:**
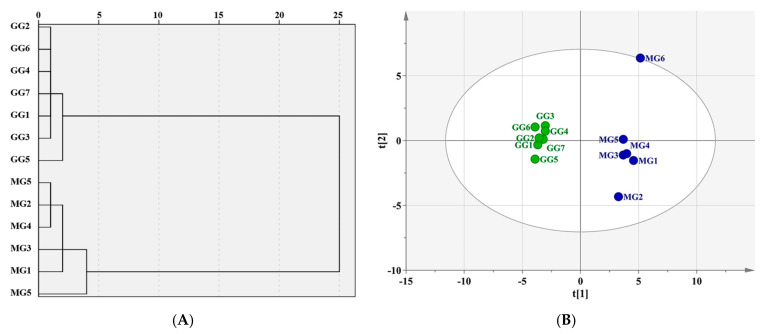
(**A**) HCA dendrogram of the 13 batches of *Ginseng*; (**B**) PCA score plots of the 13 batches of *Ginseng*.

**Figure 3:**
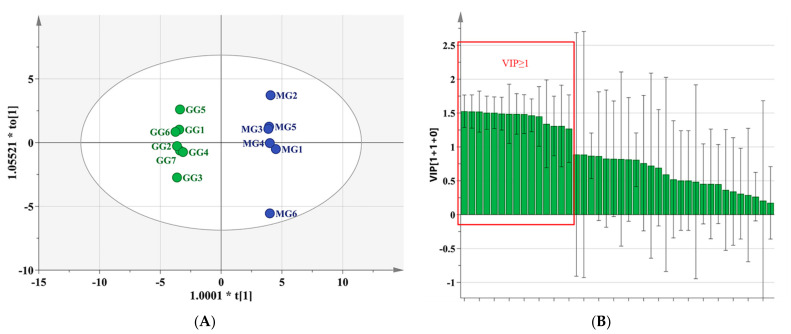
(**A**) OPLS-DA score plots of the 13 batches of *Ginseng*; (**B**) The value of VIP of constituents (* *p* < 0.05).

**Figure 4:**
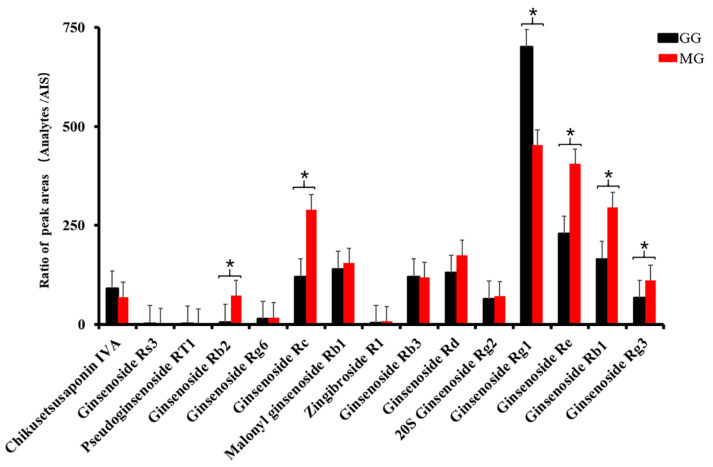
*t*-test analysis results of the 15 identified components (* *p* < 0.05).

As seen in the dendrogram Figure 2A, 13 batches of *Ginseng* from Liaoning could be divided into two categories using HCA, of which GG was clustered into one group, and MG into another. Moreover, the PCA score plot illustrated that there was an efficacious separation between groups G1–G7 and M1–M6, as shown as Figure 2B, which was consistent with the results of HCA. In order to clarify the characteristic components that contributed greatly to this distinction, OPLS-DA was applied. The score plot, as shown in Figure 3A, indicated that the two groups were highly distinct with R^2^Y = 0.999 and Q^2^ = 0.973, and 15 components demonstrated VIP ≥ 1, as shown in Figure 3B. However, among them, only six constituents—ginsenosides Rg1, Re, Rb1, Rc, Rb2, and Rg3—revealed significant differences between GG and MG (* *p* < 0.05) according to the results of a Student’s *t*-test, as shown in Figure 4.

As above, all of the 15 chemometric components were common ingredients of GG and MG. Six ginsenosides were established as the qualitative and quantitative markers for in vitro identification of GG and MG. It was concluded that these six ingredients could be considered as preliminary marker candidates to distinguish GG from MG. Although these six components had significant differences in content, this seldom indicates the quality of TCMs. The selected markers, determined via efficacy mining, were considered to be more representative of quality.

### 2.3. Comparative Study of the Pharmacodynamics of GG and MG in Paclitaxel-Induced Liver Injury

To further explore the discriminating markers of GG and MG underlying their respective pharmacodynamics, we conducted a comparative study of their liver-protective efficacies using a paclitaxel-induced liver injury rat model. Batches of GG5 and GG7, and MG1 and MG4, were included in the contrastive pharmacodynamic analysis as the contents of the 15 chemometric components in these batches were close to their average values in GG and MG, respectively.

**Figure 5:**
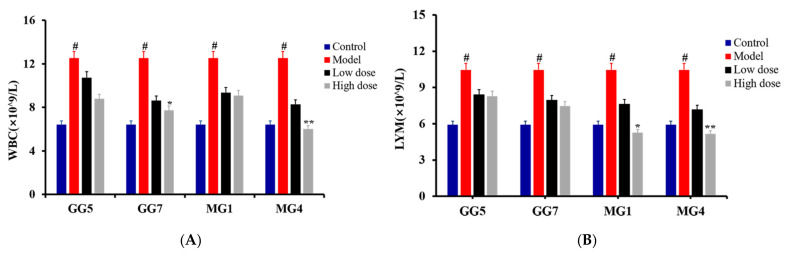
Effect of *Ginseng* on WBC (**A**) and LYM (**B**) in the blood of rats with liver injury induced by paclitaxel (compared with control group, # *p* < 0.05; compared with model group, * *p* < 0.05 and ** *p* < 0.01).

**Figure 6:**
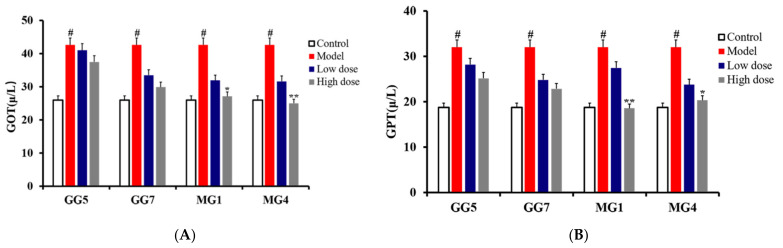
Effect of *Ginseng* on the activity of GOT (**A**) and GPT (**B**) in the serum of rats with liver injury induced by paclitaxel (compared with control group, # *p* < 0.05; compared with model group, * *p* < 0.05 and ** *p* < 0.01).

As shown in Figure 5, an obvious elevation in white blood cell count (WBC) and lymphocyte count (LYM) could be observed in the model group compared with the control group (# *p* < 0.05), indicating abnormal regulation of autoimmunity and the development of inflammation. Furthermore, expression of the liver function indicators glutamic-oxaloacetic transaminase (GOT) and glutamic-pyruvic transaminase (GPT) were also increased markedly in the model group compared with the control group, as shown as Figure 6 (# *p* < 0.05), suggesting a decrease in liver function and the occurrence of parenchymal hepatic disease [25,26,27]. Through the above, it could be concluded that the liver injury model was successfully established. These escalating alterations were all consistently restored after treatment with GG and MG; however, MG exerted stronger therapeutic effects than GG based on the results of a *t*-test (* *p* < 0.05; ** *p* < 0.01). Therefore, our research demonstrates for the first time that the hepatoprotective effects of MG are superior to those of GG through in vivo pharmacology experiments.

### 2.4. Discovery of Pharmacodynamic-Based Markers to Distinguish GG from MG

#### 2.4.1. Correlation Analysis between Characteristic Components and Pharmacodynamic Indicators

**Figure 7:**
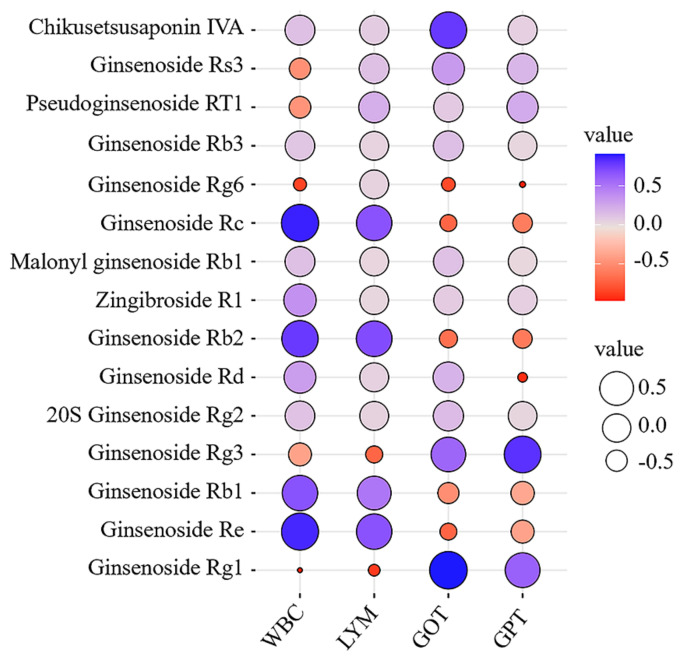
Bubble chart of Pearson correlation coefficients.

Based on pharmacological comparison, a correlation analysis of the relative peak areas of the 15 characteristic components and the levels of the four pharmacodynamic indicators was performed with the corresponding GG5, GG7, MG1, and MG4 batch sample data. Evaluation of the correlation degree by the absolute value of the Pearson correlation coefficient: 0.6 ≤ |γ| ≤ 1 indicated correlation. Ginsenosides Re, Rb1, Rc, and Rb2 were positively correlated with WBC and LYM, while ginsenosides Rg1 and Rg3 were positively correlated with GOT and GPT, as shown in Figure 7 and Table S1, indicating that ginsenosides Rg1, Re, Rb1, Rc, Rb2, and Rg3 could participate in regulation of the milieu interne in morbid state and represent candidate markers for the distinction of GG from MG based on their underlying pharmacodynamics.

#### 2.4.2. Identification of Effective Representative Substances of GG and MG Using Bioinformatic Analysis

Bioinformatic analysis was applied to visualize the large-scale data of “components–targets–pathways” and for virtual excavation of the bioactive markers capable of differentiating GG from MG, in consideration of the “multi components-multi targets” characteristic of TCMs.

**Figure 8:**
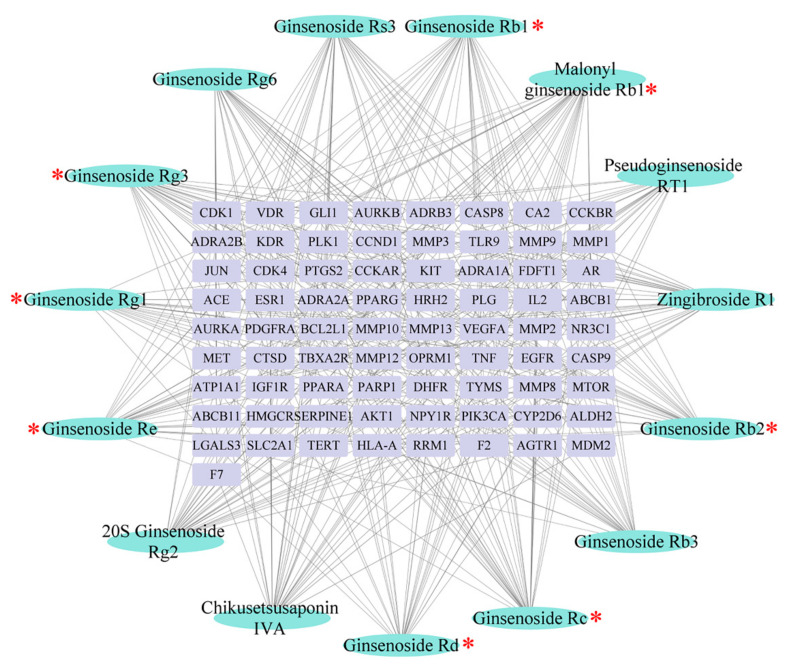
“Ingredient–target” network diagram (blue, ingredient; purple, target), * indicates ingredients with a higher than the average degree value (mean value = 27.4).

**Figure 9:**
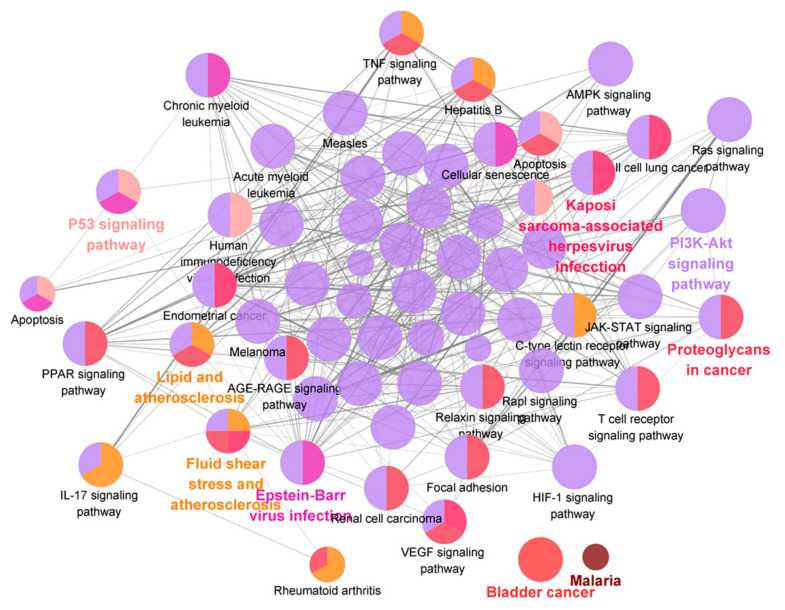
Pathway enrichment analysis of 15 characteristic components using Cytoscape.

Target genes of the 15 characteristic components were predicted by the SwissTargetPrediction database. Pathway enrichment analysis was performed with Cytoscape. A total of 73 targets and 62 pathways with significant differences were obtained. As shown in Figure 8 and Figure 9, *Ginseng* mainly participated in the regulation of inflammation and liver function through modulation of the pathways of PI3K-Akt, Ras, HIF-1, TNF, PPAR, AMPK, and p53, in addition to various pathways related to bile acid and lipid metabolism. These crucial targets and pathways were involved in the anti-liver injury effects of *Ginseng* [28,29,30,31,32,33]. Additionally, the degree value is a key parameter to assess the interconnectedness of components and their corresponding symptoms; the degree values of ginsenosides Rg1, Re, Rb1, Rc, Rb2, Rd, and Rg3, as well as malonyl ginsenoside Rb1, were higher than the average degree value (mean value = 27.4; as shown as Table S2).

Integrating all above results, the ginsenosides Rg1, Re, Rb1, Rc, Rb2, and Rg3 could be regarded as effective markers for distinguishing GG from MG. Moreover, in consideration of previous studies, it has been widely reported that ginsenoside are the most important components of *Ginseng* contributing to its beneficial properties. Ginsenosides Rg1, Re, and Rg3 act on the PI3K–Akt signaling pathway to reduce oxidative stress response [34,35,36]. In addition, ginsenosides Rb1, Rc, and Rb2 possess anti-inflammatory activities through the inhibition of both MAPK signaling pathways and the expression of TNF [37,38,39]. In agreement, our research identified these six ginsenoside compounds as pharmacodynamic-based markers to distinguish GG from MG.

## 3. Materials and Methods

### 3.1. Chemicals and Reagents

A total of 13 batches of *Ginseng* collected from Liaoning (including 7 batches of GG and 6 batches of MG) of different ages were obtained from Tong-Ren-Tang TCMs store (Shenyang, China). HPLC-grade acetonitrile, methanol, and formic acid were all purchased from Fisher Scientific (Fair Lawn, NJ, USA); all other reagents were analytical grade (Shandong Yuwang Industrial Co., Ltd., Yucheng, China). The GOT/GPT commercial enzyme-linked immunosorbent assay (ELISA) kits were purchased from the Nanjing Jiancheng Institute of Biological Engineering (Nanjing, China).

### 3.2. HPLC Q-TOF/MS Analysis

#### 3.2.1. Sample Preparation

*Ginseng* powder (1 g, passed through a 5-mesh sieve) was extracted under reflux with 10 times the volume of water for 1 h, and the progress was repeated twice. A solution with concentration 50 mg/mL (medicinal material dosage/volume) was obtained by combining the extracts. Then, the solution was centrifuged at 12,000 rpm for 10 min and filtered through a 0.22 μm filter membrane for HPLC Q-TOF/MS analysis. Appropriate amounts of the reference substance ginsenosides Re, Rf, Rb1, Rc, F1, Ro, Rb2, Rd, Rg3, F2, 20R-ginsenoside Rh1, and chikusetsusaponin Iva (ShanghaiyuanyeBio-Technology Co., Ltd., Shanghai, China) were weighed and dissolved in methanol to prepare a mixed reference solution containing the 12 chemical components. Digoxin (25 ng/mL, Chengdu Chroma-Biotechnology Co., Ltd., Sichuan, China) was used as an IS for semiquantitative analysis. All samples were kept at 4 °C during analysis.

#### 3.2.2. HPLC Q-TOF/MS Conditions

Analyses were performed on a 1260 Infinity HPLC system (Agilent Technology, Santa Clara, CA, USA). A reverse-phase column of Phenomenex Kinetex XB C18 (100 mm × 4.6 mm, 2.6 μm) was used for the chromatographic separations with a flow rate of 0.5 mL/min at 30 °C. The mobile phase consisted of 0.1% formic acid-water (A) and 0.1% formic acid-acetonitrile (B) with a gradient procedure as follows: 0–13 min, 5–30% B; 13–27 min, 30–40% B; 27–30 min, 40–70% B; 30–31 min, 70–90% B; 31.1–31.1 min, 90–5% B; 31.1–35 min, 5–5% B. The injection volume of each sample was 5 µL and the temperature of the sample plate was maintained at 4 °C.

A quadrupole time-of-flight mass spectrometry system (Triple TOF 5600, AB SCIEX Corporation, Foster City, CA, USA) was used to obtain the total ion chromatograms (TIC) in negative electrospray ionization (ESI) mode. The optimal instrument parameters were as follows: spray voltage of 4500 V, a source heater temperature of 550 °C, the range of *m*/*z* set to 100–2000 and nitrogen was used as the atomizing gas and other auxiliary gases. PeakView software (version 1.2.1, SCIEX) was used for data analysis and the precision error threshold was fixed at 5 ppm.

### 3.3. Animals

Healthy Sprague-Dawley rats (200–220 g; 8 weeks old; NO.SCXK(Liao)2020---0001) were provided by the Experimental Animal Center of Shenyang Pharmaceutical University and housed in a specialized pathogen free (SPF) standard environment (ambient temperature 22 ± 2 °C, with relative humidity of 50 ± 10 % and a natural light-dark cycle). All experiments complied with the Animal Experiment Code of Shenyang Pharmaceutical University and approval was obtained from the animal ethics committee of the institution (Ethical Approval number: SYPU-IACUC-G2P-2021-68). After a week of domestication of the rats, they were randomly divided into six groups: control, model, and both low (300 mg/kg) and high doses (800 mg/kg) of the 2 batches of GG (GG5/GG7) and 2 batches of MG (MG1/MG4) (*n* = 6). Food and water were available ad libitum.

Paclitaxel (10 mg/kg, Haikou Pharmaceutical Factory Co., Ltd, Hainan, China) was given intraperitoneally to induce liver injury once a day for two weeks. In the meantime, the rats in the medication administration groups were gavaged with the predetermined dose of *Ginseng* 1 h after the injection of paclitaxel, while rats in the control and model groups received the same volume of normal saline. One hour after the final administration, two blood samples (each 1.5 mL, one sample transferred into the tubes with anticoagulants directly, the other transferred into the heparinized tubes to obtain serum samples) were collected from the suborbital vein of each rat and stored at −80 °C until required.

### 3.4. Detection of Pharmacodynamic Indicators

In order to detect the levels of blood routine parameters WBC and LYM, a 1.5 mL blood sample of each rat was measured with an automatic animal blood cell analyzer (BC2800Vet Shenzhen).

Serum samples were used to measure the expression of liver function indicators, including GOT and GPT, according to the manufacturer’s instructions.

### 3.5. Bioinformatics Analysis Process

Network pharmacology analysis was performed with the characteristic ingredients in this study. The PubChem (https://pubchem.ncbi.nlm.nih.gov/ accessed on 30 July 2021) and SwissTargetPrediction (http://www.swisstargetprediction.ch/ accessed on 30 July 2021) databases were integrated to obtain the corresponding targets of each ingredient. The targets closely associated with “liver injury” were retrieved from the Therapeutic Target (http://db.idrblab.net/ttd/ accessed on 30 July 2021), GeneCards (https://www.genecards.org/ accessed on 30 July 2021), and Comparative Toxicogenomics Databases (http://ctdbase.org/ accessed on 30 July 2021). Finally, Cytoscape (3.8.2, USA) software was used to visualize the “component–target–pathway” network.

### 3.6. Statistical and Data Analysis

All values are presented as means ± SD. The data were analyzed using SIMCA14.0 (UmetricsAB, Umea, Sweden) and SPSS (SPSS Inc., Chicago, IL, USA). Data from two groups were analyzed using a two-tailed Student’s *t*-test and *p* values lower than 0.05 were considered significant.

## 4. Conclusions

In the present study, an integrated mutually oriented “chemical profiling–pharmaceutical effect” strategy was proposed and successfully applied to identify distinct bioactive markers for the identification of GG and MG for the first time. First, we established a chemical component database of *Ginseng* using HPLC Q-TOF/MS, which contained 40 compounds from GG and 41 compounds from MG. Multiple chemometric methods were further applied to discover the characteristic components. We then conducted a comparative study of the pharmacodynamics of GG and MG, which indicated that MG may exert superior therapeutic effects to GG. Furthermore, in order to explore the components responsible for this difference in efficacy, correlation analysis combined with network pharmacology technology was applied. Ginsenosides Rg1, Re, Rb1, Rc, Rb2, and Rg3 were identified as markers underlying the difference in hepatoprotective efficacy which can be used to distinguish GG from MG. These findings may contribute to the development of quality control and thus enhance the clinical efficacy of *Ginseng*.

## Figures and Tables

**Table 1:** A summary of the tested samples of *Ginseng*.

NO.	Origins	Years
MG1	Liaoning, Benxi	8
MG2	Liaoning, Benxi	8–9
MG3	Liaoning, Huanren	14
MG4	Liaoning, Huanren	17
MG5	Liaoning, Shenyang	10
MG6	Liaoning, Shenyang	15
GG1	Liaoning, Jinzhou	4
GG2	Liaoning, Fushun	4
GG3	Liaoning, Jinzhou	5
GG4	Liaoning, Fushun	5
GG5	Liaoning, Jinzhou	6
GG6	Liaoning, Jinzhou	4
GG7	Liaoning, Fushun	6

**Table 2:** Detected compounds in *Ginseng* by HPLC Q-TOF/MS.

No.	Identification	Formula	Adduct Ion	*m*/*z*	Error (ppm)	Retention Time (min)	Fragment Ions (*m*/*z*)
1	Pseudogisnenoside RT2	C_41_H_70_O_14_	[COOH]^+^	831.4726	−1.3	14.45	161.0451, 491.3708, 653.4280, 785.4686
2	Floralginsenoside A	C_42_H_72_O_16_	[H]^−^	831.4726	−2.7	14.51	161.0451, 491.3708, 653.4280, 785.4686
3	Notoginsenoside R3	C_48_H_82_O_19_	[COOH]^+^	1007.542	−0.5	15.03	637.4349, 781.4764, 799.4853, 961.5411
4	Notoginsenoside R1	C_47_H_80_O_18_	[COOH]^+^	977.5311	−0.5	15.34	131.0352, 475.3777, 637.4310, 799.4866
5	Ginsenoside Rg1	C_42_H_72_O_14_	[COOH]^+^	845.4898	0.5	15.87	161.0455, 475.3782, 637.4317, 799.4862
6	Ginsenoside Re	C_48_H_82_O_18_	[COOH]^+^	991.5478	0.5	16.07	161.0462, 179.0564, 475.3810, 619.4249
7	Pseudoginsenoside RT3	C_41_H_70_O_13_	[COOH]^+^	815.4792	0.6	17.82	391.2720, 475.3806, 553.3349, 637.4354
8	4’-O-acetylpseudoginsenoside F11	C_44_H_74_O_15_	[COOH]^+^	887.5006	0.8	17.92	161.0434, 391.2863, 475.3884, 619.4274
9	6’-O-acetylginsenoside Rg1	C_44_H_74_O_15_	[COOH]^+^	887.5014	1.8	19.22	475.3800, 619.4013, 637.4266, 781.4766
10	Pseudoginsenoside F11 *	C_42_H_72_O_14_	[H]^−^	799.485	0.2	20.21	161.0455, 415.3218, 491.3768, 653.4331
11	Ginsenoside Rf	C_42_H_72_O_14_	[COOH]^+^	845.4899	0.6	20.57	161.0468,415.3225, 653.4201, 799.4903
12	Notoginsenoside R2	C_41_H_70_O_13_	[COOH]^+^	815.4793	0.7	21.49	475.3778, 619.4207, 637.4388, 769.4760
13	Ginsenoside F3	C_41_H_70_O_13_	[COOH]^+^	815.4793	0.7	21.58	161.0463, 457.3778, 475.3778, 619.4207
14	Ginsenoside Ra2 **	C_58_H_98_O_26_	[H]^−^	1209.6292	1.5	21.83	149.0419, 323.1010, 621.4420, 783.4917
15	Ginsenoside Rb1	C_54_H_92_O_23_	[COOH]^+^	1153.599	−0.9	22.50	161.0469, 221.0674, 459.3756, 621.4323
16	20S Ginsenoside Rg2	C_42_H_72_O_13_	[COOH]^+^	829.4951	0.8	22.67	391.2899, 475.3792, 619.4224, 637.4360
17	Malonyl ginsenoside Rb1	C_57_H_94_O_26_	[H]^−^	1193.595	−1.1	22.93	179.0562, 783.4874, 927.5361, 945.5423
18	20S Ginsenoside Rh1	C_36_H_62_O_9_	[COOH]^+^	683.4369	0.6	22.94	391.2749, 457.3619, 475.3830, 637.4316
19	Ginsenoside Ra1 **	C_58_H_98_O_26_	[H]^−^	1209.6282	0.7	23.08	621.4382, 783.4733, 945.5652, 1077.5902
20	Ginsenoside Rc	C_53_H_90_O_22_	[COOH]^+^	1123.59	0	23.44	149.0461, 621.4406, 765.4835, 783.4940
21	Ginsenoside Rb3	C_53_H_90_O_22_	[COOH]^+^	1123.589	−0.9	23.85	621.4402, 783.4904, 945.5373, 1077.5851
22	Ginsenoside F1	C_36_H_62_O_9_	[COOH]^+^	683.4365	−0.1	23.91	161.0456, 391.2859, 475.3801, 637.4345
23	Malonyl ginsenoside Rc	C_56_H_92_O_25_	[H]^−^	1163.584	−0.9	23.94	783.4904, 945.5373, 1059.5737, 1077.5851
24	Ginsenoside Ro	C_48_H_76_O_19_	[COOH]^+^	1001.496	0.9	24.4	455.3498, 523.3764, 569.3854, 613.3544
25	Ginsenoside Rb2	C_53_H_90_O_22_	[COOH]^+^	1123.59	0.2	24.8	621.4420, 783.4967, 945.5501, 1077.5889
26	Malonyl ginsenoside Rb2	C_56_H_92_O_25_	[H]^−^	1163.584	−0.9	24.83	459.3823, 621.4387, 783.4917, 945.5454
27	Quinquenoside R1	C_56_H_94_O_24_	[COOH]^+^	1195.609	−0.9	25.05	179.0564, 323.0999, 621.4222, 783.4717
28	Ginsenoside Rs1	C_55_H_92_O_23_	[H]^−^	1119.593	−2.1	25.4	1077.5807, 945.5515, 783.4877, 621.4542
29	Pseudoginsenoside RT1	C_47_H_74_O_18_	[H]^−^	925.4797	−0.5	25.48	523.4065, 569.3840, 613.3785, 701.4410
30	20R Ginsenoside Rh1	C_36_H_62_O_9_	[COOH]^+^	683.437	0.7	25.89	391.2854, 457.3704, 475.3805, 637.4319
31	Ginsenoside Rd	C_48_H_82_O_18_	[COOH]^+^	991.5474	0.2	26.65	161.0465, 621.4410, 783.4907,945.5418
32	Malonyl ginsenoside Rd	C_51_H_84_O_21_	[H]^−^	1031.543	−0.6	26.97	621.4394, 765.4816, 783.4937, 927.5336
33	Pseudoginsenoside RC1	C_50_H_84_O_19_	[COOH]^+^	1033.552	−5.4	27.44	161.0453, 459.3802, 621.4449, 765.4792
34	Zingibroside R1	C_42_H_66_O_14_	[H]^−^	793.4373	−0.8	27.45	455.3508, 569.3859, 613.3767, 631.3851
35	Ginsenoside Rg10	C_42_H_70_O_13_	[COOH]^+^	827.4781	−0.8	29.98	161.0415, 221.0695, 619.4169, 781.4760
36	Ginsenoside Rg6	C_42_H_70_O_12_	[COOH]^+^	811.4842	0.5	31.86	161.0447, 457.3617, 601.4126, 619.4218
37	Ginsenoside Rg3	C_42_H_72_O_13_	[COOH]^+^	829.4949	0.6	32.10	161.0468, 459.3864, 621.4404,783.4958
38	Ginsenoside Rk3	C_36_H_60_O_8_	[COOH]^+^	665.4262	0.4	32.37	161.0457, 457.3588, 619.4184, 665.4284
39	Chikusetsusaponin IVA	C_42_H_66_O_14_	[H]^−^	793.438	0	32.59	455.3530, 569.3874, 613.3763, 793.4408
40	Ginsenoside F2	C_42_H_72_O_13_	[COOH]^+^	829.4949	0.6	32.73	161.0460, 459.3853, 621.4396,783.4938
41	Ginsenoside Rs3	C_44_H_74_O_14_	[COOH]^+^	871.5011	−4.4	32.92	161.0461, 375.2889, 459.3834, 621.4326
42	Ginsenoside Rk1	C_42_H_70_O_12_	[COOH]^+^	811.4845	0.8	34.20	161.0460, 537.3988, 603.4275, 765.4791

* indicates ingredients belong to GG; ** indicates ingredients belong to MG.

## Data Availability

Not applicable.

## Supplementary Materials

The following are available online, Figure S1: (**A**) Extracted ion chromatograms of 12 reference substances: 1. Ginsenoside Re; 2. Ginsenoside Rf; 3. Ginsenoside Rb1; 4. Ginsenoside Rc; 5. Ginsenoside F1; 6. Ginsenoside Ro; 7. Ginsenoside Rb2; 8. 20R Ginsenoside Rh1; 9. Ginsenoside Rd; 10. Ginsenoside Rg3; 11. Chikusetsusaponin IVa; 12. Ginsenoside F2; (**B**) The total ion chromatograms of GG5; (**C**) The total ion chromatograms of MG1, Table S1: Pearson correlation coefficient between the 15 characteristic ingredients and 4 pharmacodynamic indexes, Table S2: The degree value of 15 characteristic components.

## Sample Availability

Samples of the compounds are available from the authors.
