# Supplementary material for: An Integrated Mutually Oriented “Chemical Profiling–Pharmaceutical Effect” Strategy for Screening Discriminating Markers of Underlying Hepatoprotective Effects to Distinguish Garden-Cultivated from Mountain-Cultivated Ginseng
Source: Molecules. 2021 Sep 8;26(18):5456. doi: 10.3390/molecules26185456 (PMC8466359; doi:10.3390/molecules26185456)
Supplement: Supplementary file 1 [file molecules-26-05456-s001.zip › molecules-1341413-supplementary.pdf]

Supporting Materials

An Integrated Mutually Oriented "Chemical Profiling–Pharmaceutical Effect" Strategy for Screening Discriminating Markers of Underlying Hepatoprotective Effects to Distinguish Garden-Cultivated from Mountain-Cultivated *Ginseng*

Saiyu Li †, Yiwen Zhang †, Panpan Yang, Minghui Tong, Luwen Xing, Qian Zhang, Kaishun Bi and Qing Li *

School of Pharmacy, Shenyang Pharmaceutical University, Shenyang 110016, China; Saiyu_Li@outlook.com (S.L.); zzzyyywen@outlook.com (Y.Z.); Yang_panpan0112@163.com (P.Y.); tongminghui0930@163.com (M.T.); xlw_aikoaikko@163.com (L.X.); zhangqian@syphu.edu.cn (Q.Z.); kaishunbi.syphu@gmail.com (K.B.)

* Correspondence: lqyxm@hotmail.com; Tel.: +86-2423986012

† These authors contributed equally to this work.

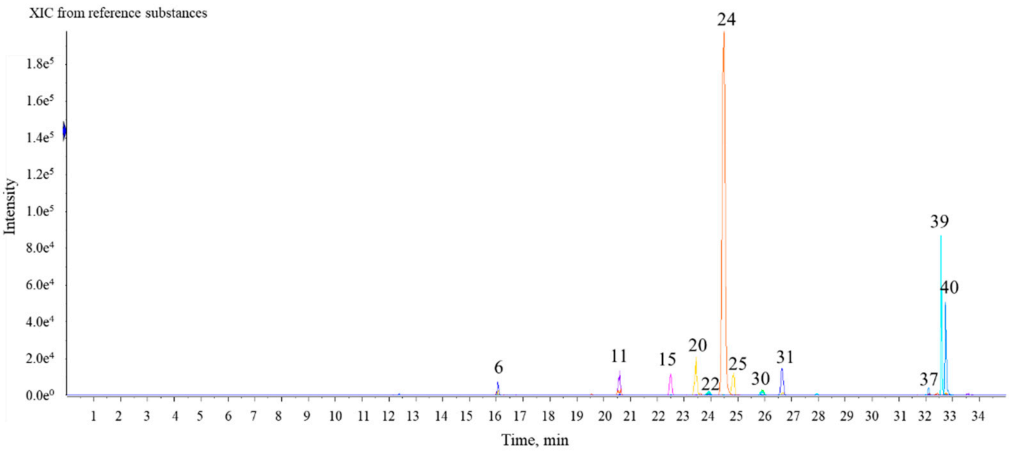

(A)

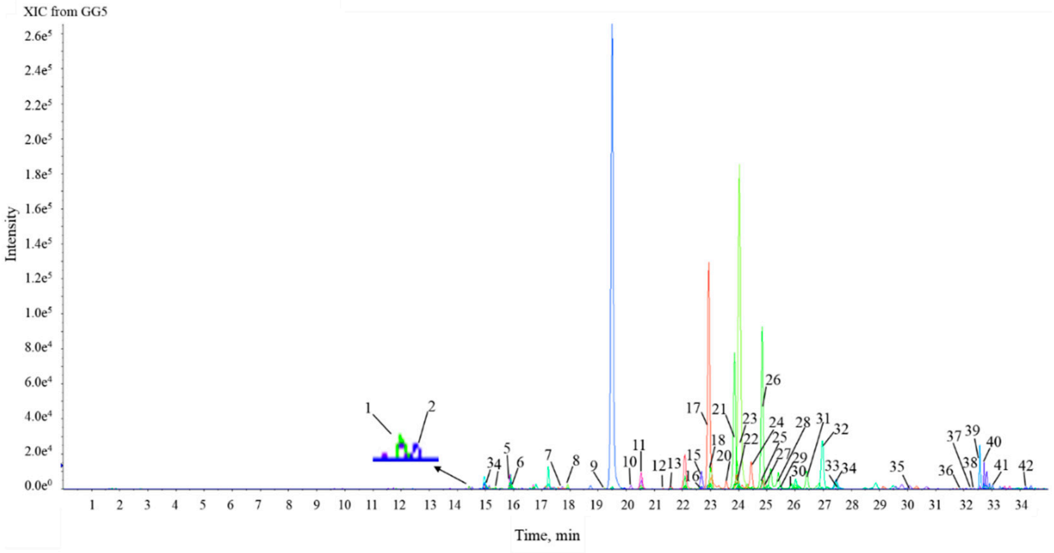

(B)

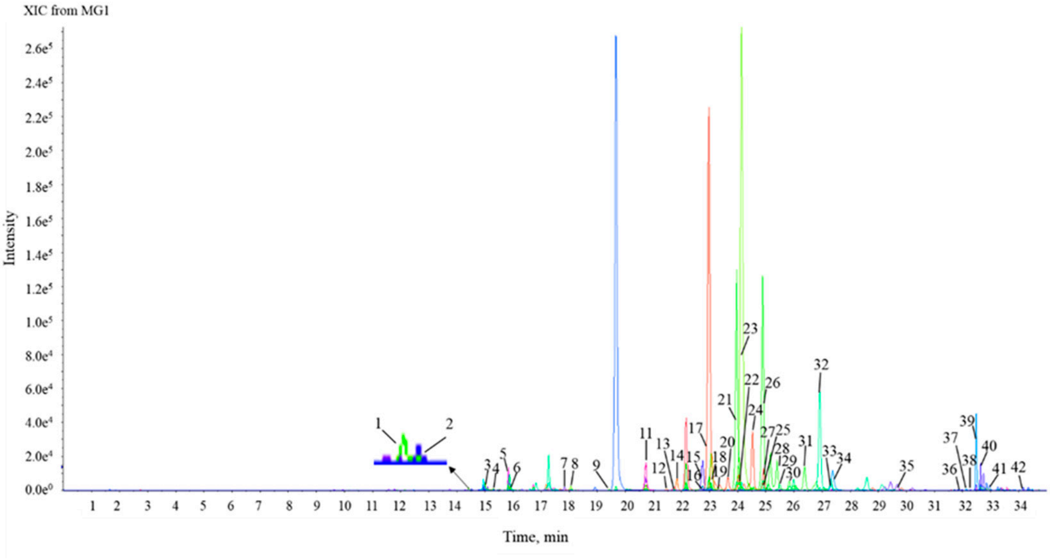

(**C**)

Figure S1. (**A**) Extracted ion chromatograms of 12 reference substances: 6. Ginsenoside Re; 11. Ginsenoside Rf; 15. Ginsenoside Rb1; 20. Ginsenoside Rc; 22. Ginsenoside F1; 24. Ginsenoside Ro; 25. Ginsenoside Rb2; 30. 20R Ginsenoside Rh1; 31. Ginsenoside Rd; 37. Ginsenoside Rg3; 39. Chikusetsusaponin IVA; 40. Ginsenoside F2; (**B**) Extracted ion chromatograms of GG5; (**C**) Extracted ion chromatograms of MG1.

Table S1. Pearson correlation coefficient between the 15 characteristic ingredients and 4 pharmacodynamics indexes.

Ingredient	Blood routine parameter		Liver function index	
	**WBC**	**LYM**	**GOT**	**GPT**
Chikusetsusaponin IVA	0.129	0.072	0.784	0.058
Ginsenoside Rs3	-0.492	0.13	0.321	0.183
Pseudoginsenoside RT1	-0.471	0.222	0.08	0.243
Ginsenoside Rb3	0.103	0.038	0.136	0.021
Ginsenoside Rg6	-0.853	0.04	-0.833	-0.967
Ginsenoside Rc*	0.877	0.673	-0.716	-0.595
Malonyl ginsenoside Rb1	0.133	0.025	0.127	0.016
Zingibroside R1	0.362	0.023	0.077	0.056
Ginsenoside Rb2*	0.78	0.702	-0.664	-0.612
Ginsenoside Rd	0.305	0.045	0.198	-0.93
20S Ginsenoside Rg2	0.116	0.043	0.157	0.028
Ginsenoside Rg3*	-0.394	-0.71	0.683	0.817
Ginsenoside Rb1*	0.676	0.603	-0.506	-0.37
Ginsenoside Re*	0.855	0.673	-0.716	-0.391
Ginsenoside Rg1*	-0.969	-0.888	0.905	0.604

"*" indicates ingredients that have a good correlation with pharmacodynamics indexes.

Table S2. The degree value of 15 characteristic components.

NO.	Component	Degree value
1	Ginsenoside Rb1	31
2	Ginsenoside Rb2	30
3	Ginsenoside Rc	30
4	Ginsenoside Re	30
5	Ginsenoside Rg1	30
6	Ginsenoside Rg3	29
7	Ginsenoside Rd	29
8	Malonyl ginsenoside Rb1	29
9	Chikusetsusaponin IVA	27
10	Ginsenoside Rg6	27
11	Ginsenoside Rs3	27
12	Ginsenoside Rb3	24

13	Pseudoginsenoside RT1	23
14	Zingibroside R1	23
15	20S Ginsenoside Rg2	22
